# Motion correction for free breathing quantitative myocardial t_2_ mapping: impact on reproducibility and spatial variability

**DOI:** 10.1186/1532-429X-17-S1-W5

**Published:** 2015-02-03

**Authors:** Sébastien Roujol, Tamer A Basha, Sebastian Weingartner, Mehmet Akcakaya, Sophie Berg, Warren J Manning, Reza Nezafat

**Affiliations:** 1 Department of Medicine (Cardiovascular Division), BIDMC / Harvard Medical School, Boston, MA, USA; 2 Computer Assisted Clinical Medicine, University Medical Center Mannheim, Heidelberg University, Mannheim, Germany; 3 Radiology, BIDMC / Harvard Medical School, Boston, MA, USA

## Background

Quantitative myocardial T_2_ mapping is a promising technique for *in-vivo* assessment of inflammation and edema [1]. Free breathing T_2_ mapping sequences increase the flexibility in the choice of the number of T_2_prep echoes times (TE_T2P_), but should be combined with respiratory motion correction technique [2]. In this study, we sought to evaluate the performance of the Adaptive Registration of varying Contrast-weighted images for improved TIssue Characterization (ARCTIC) algorithm [3] for in-plane motion correction in T_2_ mapping data and its impact on in-vivo reproducibility and spatial variability of myocardial T_2_ estimates.

## Methods

Seven healthy adult subjects (30±17y, 3male) were imaged using a 1.5 T Phillips scanner. T_2_ mapping was performed using either 1) a "T_2P_4TE" sequence (4 T_2_prep echo times=[0, 25, 50, ∞]), or 2) a "T_2P_20TE" sequence (20 T_2_prep echo times=[0, 25, 30, 35, …, 95, 100, ∞, ∞, ∞]) [4]. TE_T2P_=∞ was simulated by acquiring an image immediately after a saturation pulse [4]. Each subject was imaged using eight T_2_ mapping scans in the following order: 1) breath-held T_2P_4TE (BH), 2) free breathing T_2P_4TE *without* respiratory navigator (FB), 3) free breathing T_2P_4TE *with* respiratory navigator (FB+NAV), and 4) free breathing T_2P_20TE *with* respiratory navigator (5 repetitions). The same 2D short axis slice was acquired with all scans using single-shot ECG-triggered acquisitions with balanced SSFP imaging readout (TR/TE/α=2.7ms/1.35ms/85°, FOV=240×240mm^2^, resolution=2.5×2.5×8mm^3^, 10 linear ramp-up pulses, SENSE rate=2, 51 phase encoding lines, linear ordering). Accuracy of in-plane motion correction was evaluated in the first three scans by measurements of the DICE similarity coefficients (DSC) (1: ideal registration, 0: none) and the myocardial boundary error (MBE) with and without using ARCTIC. T_2_ mapping reproducibility and spatial variability with and without using ARCTIC was evaluated over the entire myocardium using the 5 repetitions of the T_2P_20TE sequence and 1) a subset of 4 T_2_prep echo times=[0ms, 25ms, 50ms, ∞] (referred to as 4TE ) and 2) all 20 T_2_prep echo times (referred to as 20TE).

## Results

ARCTIC increased DSC in BH data (0.90±0.02 vs. 0.87±0.05, p=0.09), FB data (0.91±0.02 vs. 0.79±0.15, p=0.009), and FB+NAV data (0.90±0.02 vs. 0.86±0.08, p=0.039), and reduced MBE in BH data (0.63±0.09 vs. 0.74±0.12, p=0.049), FB data (0.60±0.12 vs. 1.16±0.71, p=0.007), and FB+NAV data (0.61±0.13 vs. 0.83±0.28, p=0.025). ARCTIC improved the reproducibility (4TE: 5.0±2.3ms vs. 5.9±3.1ms, p=0.011; 20TE: 2.4±1.0ms vs. 4.3±3.9ms, p=0.002) and reduced spatial variability (4TE: 11.1±3.6ms vs. 13.7±4.3ms, p<0.001; 20TE: 7.9±1.8ms vs. 10.6±5.3ms, p=0.001) of in-vivo T_2_ mapping.

## Conclusions

The ARCTIC technique substantially reduces spatial mis-alignment among T_2_-weighted images and improves both the reproducibility and the spatial variability of in-vivo T_2_ mapping.

**Figure 1:**
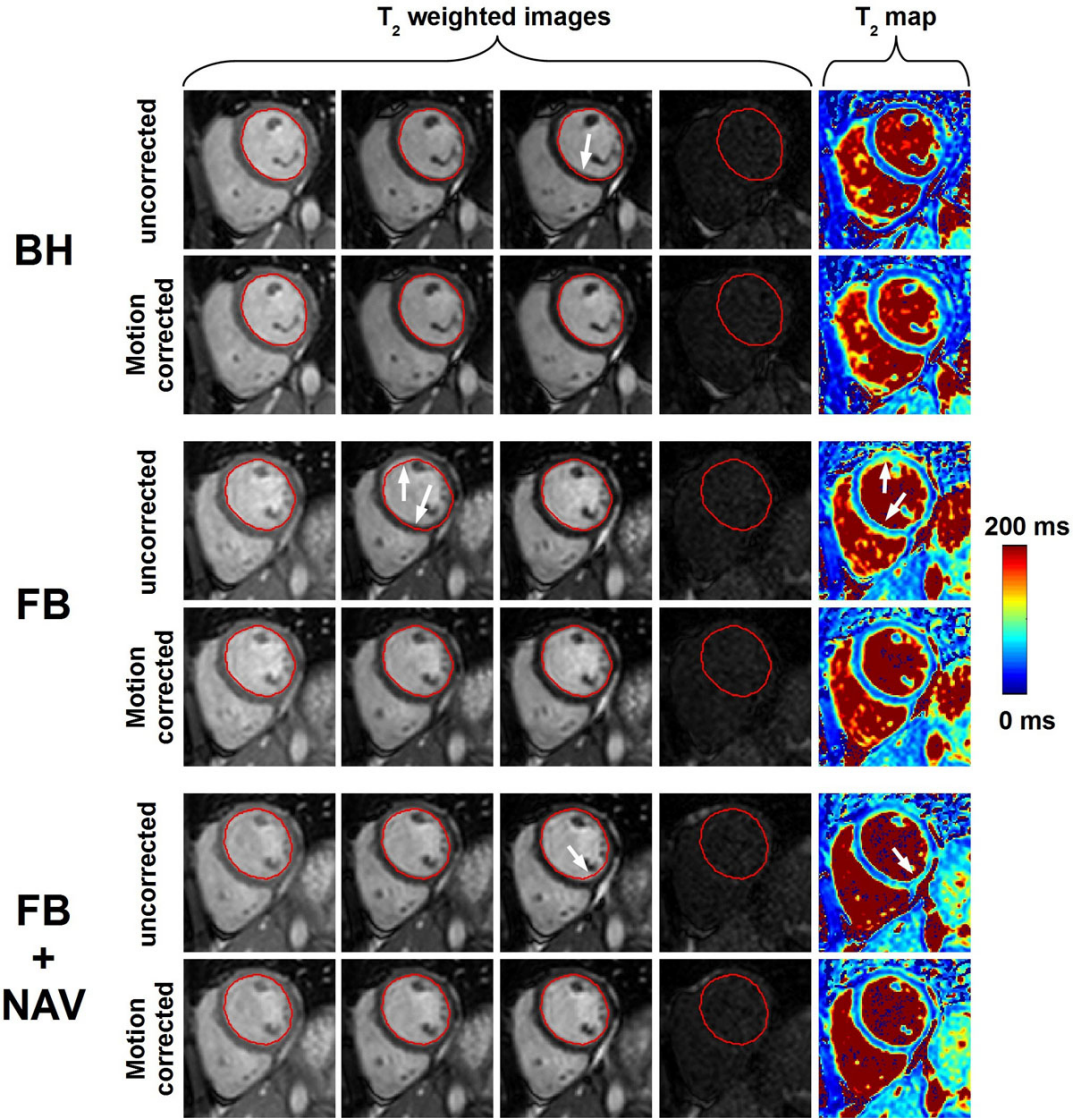
T_2_ scans from one subject acquired using the T_2P_4TE sequence under breath-hold (BH), free breathing (FB), and free breathing with respiratory navigator gating (FB+NAV). Data are shown without (uncorrected) and with (motion corrected) in-plane motion correction. The endocardial contour of the left ventricular (LV) myocardium, drawn on the reference image (1st image) of each scan, is reported in all subsequent T_2_-weighted images to facilitate visual motion assessment. Misalignments observed among uncorrected images (white arrows) were substantially reduced after in-plane motion correction using ARCTIC. Furthermore, artifacts in uncorrected T_2_ maps (white arrows) were reduced in motion corrected T_2_ maps.

**Figure 2:**
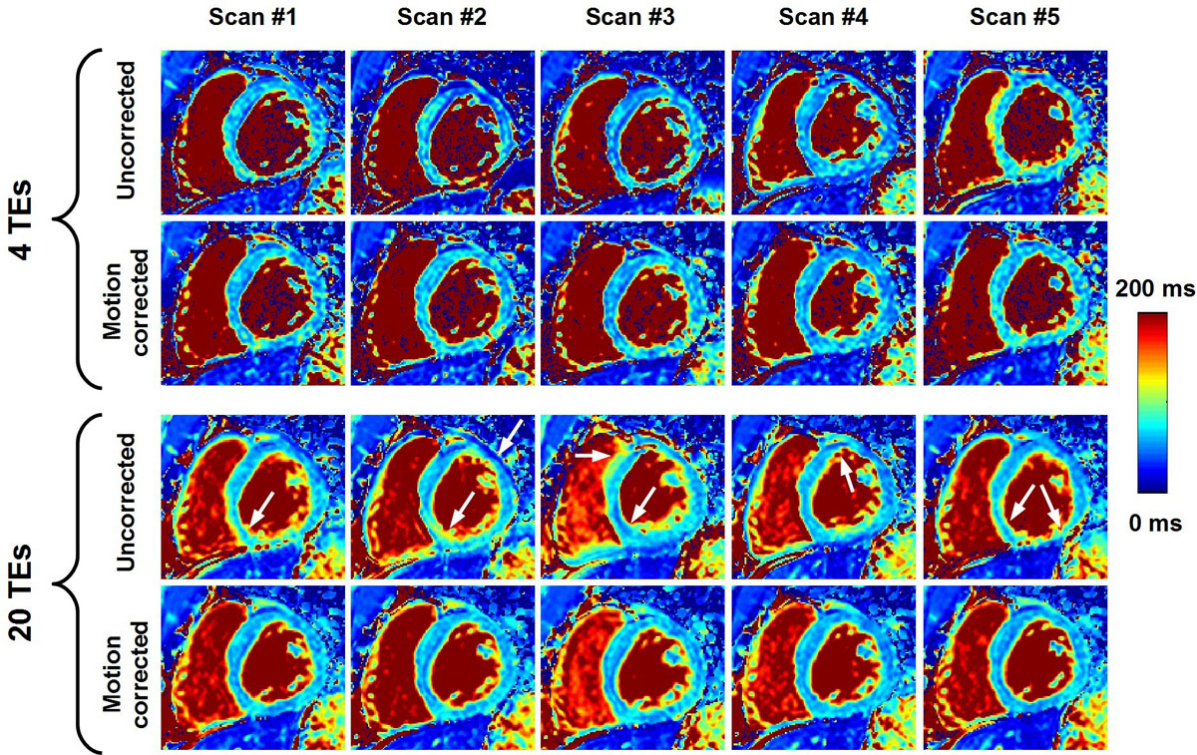
Example of multiple T_2_ maps acquired on the same subject using the five repetitions of the T_2P_20TE sequence acquired under free breathing conditions with respiratory navigator gating. T_2_ maps were reconstructed with all T_2_prep echo times (20 TEs) or only a subset of the T_2_prep echo times (0ms, 25ms, 50ms, ∞) (4 TEs). While the remaining in-plane motion generates artifacts on the directly reconstructed T_2_ maps (uncorrected), substantial improvement of T_2_ map quality was obtained using in-plane motion correction (motion corrected). As expected, the homogeneity of the T_2_ maps greatly improved when using all 20 T_2_prep echo times compared to only 4 T_2_prep echo times.
